# Case report and review of the literature: multiplanar CT as a supportive diagnostic tool in pleuroperitoneal leak during peritoneal dialysis

**DOI:** 10.3389/fmed.2026.1776767

**Published:** 2026-05-08

**Authors:** Alessia Negri, Francesco Lombardi, Giampiero Facchini, Luciana Paladini, Angelo Calabrese, Annamaria Della Corte, Fabrizio Fabiani, Lorenzo Maria Tramaglino

**Affiliations:** 1 Department of Pulmonology, Università Cattolica del Sacro Cuore, Rome, Italy; 2 Pulmonary Medicine Unit, Azienda Ospedaliera San Giovanni Addolorata, Rome, Italy

**Keywords:** case report, diaphragmatic defect, peritoneal dialysis, pleural effusion, pleuroperitoneal communication, multiplanar CT, review

## Abstract

**Introduction:**

Pleural effusion is a recognized complication of peritoneal dialysis (PD), with pleuroperitoneal communication (PPC) representing a rare but clinically significant cause. Patients typically present with dyspnea, reduced ultrafiltration volume and edema.

**Case presentation:**

A 52-year-old woman on daily peritoneal dialysis reported progressive dyspnea and chronic localized chest pain, which is an uncommon presentation. Imaging revealed a massive right-sided pleural effusion, and pleural fluid analysis showed a transudate based on Light’s criteria with markedly elevated glucose concentration. Computed tomography (CT) with multiplanar reconstruction demonstrated findings suggestive of a focal diaphragmatic discontinuity, however, the defect was not clearly visualized and no functional confirmation of pleuroperitoneal communication was achieved. Peritoneal dialysis was discontinued, and the patient was transitioned to hemodialysis (HD). The pleural effusion resolved completely in 5 days without further complications.

**Discussion:**

This case highlights the importance of considering pleuroperitoneal communication in PD patients with pleural effusion and chest pain. Due to the absence of a clearly defined gold standard, the diagnosis can be challenging. A key diagnostic marker for pleuroperitoneal leak is an elevated glucose concentration in the pleural fluid. Several imaging techniques can aid diagnosis but generally suffer from relatively low sensitivity.

**Conclusion:**

Pleuroperitoneal leak is a rare but severe complication of PD. According to our experience, CT with multiplanar reconstruction provided anatomical findings suggestive of a diaphragmatic defect, and, in addition to pleural fluid biochemical analysis, helped strengthen the diagnostic suspicion of PPC. However, further studies comparing imaging findings with surgical confirmation are needed to determine its true diagnostic accuracy and its reliability in identifying the diaphragmatic defect.

## Introduction

Peritoneal dialysis (PD) is a therapeutic option for the treatment of end-stage renal failure but may cause both infectious and non-infectious complications. Pleural effusion in patients undergoing PD can have multiple causes, including hypervolemia, parapneumonic effusions and uremic pleuritis (1). Pleuroperitoneal leak is a rare complication of PD with an incidence less than 2% (2). This phenomenon results from the transdiaphragmatic passage of dialysate into the pleural space, typically on the right side (2–4), through congenital or acquired defects in the diaphragm. It is generally an early complication, with 89% of reported cases occurring within 12 months (5), but delayed presentations have also been reported (6).

Patients may present with non-specific symptoms such as dyspnea and progressive reduction in ultrafiltration volumes, but atypical presentations have also been reported, such as syncopal episode (4). Diagnosis relies on a combination of biochemical analysis of pleural fluid—often revealing a transudate with high glucose concentration (7)—and advanced imaging techniques such as peritoneal scintigraphy or computed tomography peritoneography (CTP).

We report the case of a patient on daily peritoneal dialysis who presented with acute dyspnea and chronic localized pain. Non-contrast chest CT revealed a massive right-sided pleural effusion and pleural fluid biochemical test showed high glucose level compared to serum glucose. To further support the clinical suspicion of pleuroperitoneal communication, we performed multiplanar reconstruction of the chest CT. The pleural effusion resolved completely with the cessation of peritoneal dialysis and transition to hemodialysis (Table 1).

**Table 1:** Timeline of clinical events, diagnostic procedures, and management during the patient’s course.

Time	Event
Month 0	Start of PD.
Month 9	Onset of chronic chest pain and exertional dyspnea.
Month 12	Onset of rest dyspnea and ED admission.
Day 1 of hospitalization	Diagnosis of pleural effusion and chest drain placement.
Day 5 of hospitalization	Multiplanar reconstruction of the chest CT suggested diaphragmatic defect. PD discontinued, switch to HD.
Day 10 of hospitalization	Ultrasound evaluation showed resolution of the pleural effusion and chest drain was removed.
Subsequent months	Continued on HD without further complications.

Moreover, a narrative literature review was performed using the PubMed database, applying the following keywords: ‘pleuroperitoneal leak’, ‘pleuroperitoneal communication’, ‘computed tomography peritoneography’, and ‘pleural effusion’ in association with ‘peritoneal dialysis’. An initial comprehensive search covering the last 20 years was conducted, followed by a focused selection of studies published within the last 5 years based on their relevance to diagnostic approaches and clinical management (Table 2).

**Table 2:** Literature review of reported cases of pleuroperitoneal leak in peritoneal dialysis patients.

Author (year)	Presentation	Diagnosis	Treatment	Outcome
Cho et al. 2010 (16)	Dyspnea, right pleuritic chest pain	Chest X-ray, biochemical PF tests, peritoneal scintigraphy	PD discontinued and converted temporary to HD, VATS + pleurodesis	Resumed PD
Bae et al. 2011 (9)	Dry cough	Chest X-ray, biochemical PF tests, CT peritoneography	PD discontinuation and converted to HD	On HD
Kennedy et al. 2011 (15)	Three cases: Dyspnea Dyspnea, weight gain, lower limb edema Not specified	Chest X-ray, biochemical PF tests Chest X ray, biochemical PF tests Biochemical PF tests, imaging not specified	PD discontinuation Therapeutic thoracentesis, PD discontinued Chest drain, PD discontinued, VATS with decortication	On HD On HD On HD
Hojs et al. 2019 (40)	Reduced ultrafiltration volumes, tachydyspnea	Chest X-ray, biochemical PF tests	PD discontinued and converted temporary to HD	Resumed PD
Nakayama et al. 2019 (23)	Dyspnea	Chest X-ray, CT peritoneography	VATS with surgical correction of the defect	Resumed PD
Dang et al. 2020 (33)	Reduced ultrafiltration volumes, dyspnea, tachypnea and hypoxia	Chest X-ray, biochemical PF tests	VATS + pleurodesis	Resumed PD
Bohra et al. 2020 (14)	Dyspnea	Chest X-ray, biochemical PF tests, peritoneal scintigraphy	Therapeutic thoracentesis, PD discontinued and converted to hemodialysis	On HD
Newallo et al. 2022 (20)	Dyspnea, chest pain	Chest X-ray, biochemical PF tests, low-dose SPECT/CT in peritoneal scintigraphy	Therapeutic thoracentesis, PD discontinued, switched to HD	On HD
Wang et al. 2023 (4)	Syncopal episode, worsening fatigue, poor appetite, difficult-to-control moderate hypertension	Chest CT, biochemical PF tests	PD discontinued	On HD
Kwan et al. 2023 (6)	Dyspnea and reduced ultrafiltration volumes	Chest X-ray, biochemical PF tests, peritoneal scintigraphy	PD discontinued and converted to hemodialysis	On HD
Jonny et al. 2024 (34)	Dyspnea and dry cough	Chest X-ray, biochemical PF tests and peritoneal scintigraphy	VATS with surgical correction of the defect + pleurodesis	Resumed PD
Bhattarai et al. 2024 (41)	Dyspnea	Chest X-ray, biochemical PF tests	Therapeutic thoracentesis, PD discontinued and converted to HD	On HD
Shah et al. 2024 (27)	Dyspnea, tachypnea, reduced ultrafiltration volumes	Chest X-ray, chest CT, biochemical PF tests	Therapeutic thoracentesis, PD discontinued and converted to HD	On HD
García Romero et al. 2024 (28)	Dyspnea, hypoxia, pleuritic pain secondary to PD sessions	Chest X-ray, chest CT, methylene blue instillation test	Converted to HD	On HD
Ria et al. 2025 (19)	2 patients: Dyspnea and reduced ultrafiltration volumes	Chest X-ray, peritoneal scintigraphy + CT peritoneography	PD discontinued and converted to HD VATS with surgical correction of the defect	On HD Resumed PD
Lafrid et al. 2025 (35)	Dyspnea, reduced ultrafiltration volumes, lower limbs edema, weight gain	Chest X-ray, biochemical PF tests, CT peritoneography	VATS + pleurodesis	Resumed PD
Suyama et al. 2025 (32)	Dyspnea, persistent cough	Chest X-ray, peritoneal scintigraphy	VATS with surgical correction of the defect	Resumed PD
Present case (2025)	Exertional and rest dyspnea, chronic and localized chest pain	Biochemical PF tests, chest CT with multiplanar reconstruction	PD discontinued and converted to HD	On HD

PD, peritoneal dialysis; HD, hemodialysis; CT, computed tomography; VATS, video-assisted thoracoscopic surgery; PF, pleural fluid.

## Case presentation

A 52-year-old Caucasian woman with end-stage chronic kidney disease and a single functioning kidney on daily automated peritoneal dialysis (APD) from approximately 12 months using a portable home-based dialysis device, presented to the emergency department (ED) with progressive dyspnea and chronic localized right-sided chest pain. She reported that the chest pain had started approximately 9 months after the initiation of peritoneal dialysis and had been present for 4 months at the time of presentation. She denied fever or cough and reported that dyspnea started as exertional and then gradually worsened in rest dyspnea. She also had a medical history of myelodysplastic syndrome, vasculopathy with bilateral optic nerve stroke and hypertension. Family history was unremarkable for renal, pulmonary, or hereditary disorders. The patient reported no history of smoking or alcohol abuse.

Physical examination revealed decreased breath sounds over the entire right hemithorax, with dullness to percussion and absent vocal fremitus. Oxygen saturation was normal (95% on ambient air), and arterial blood gas analysis showed compensated metabolic acidosis (pH 7.43, PaO_2_ 79 mmHg, PaCO_2_ 27.6 mmHg, HCO_3_^–^17.9 mmol/L, BE −5.4 mmol/L, Lactate 1.25 mmol/L). Blood tests demonstrated mild anemia (Hemoglobin 9.5 g/dL), elevated serum creatinine (9.8 mg/dL), and normal inflammatory markers. Electrocardiogram and bedside echocardiography excluded acute cardiac pathology, showing preserved ejection fraction and no evidence of pericardial effusion.

**Figure 1:**
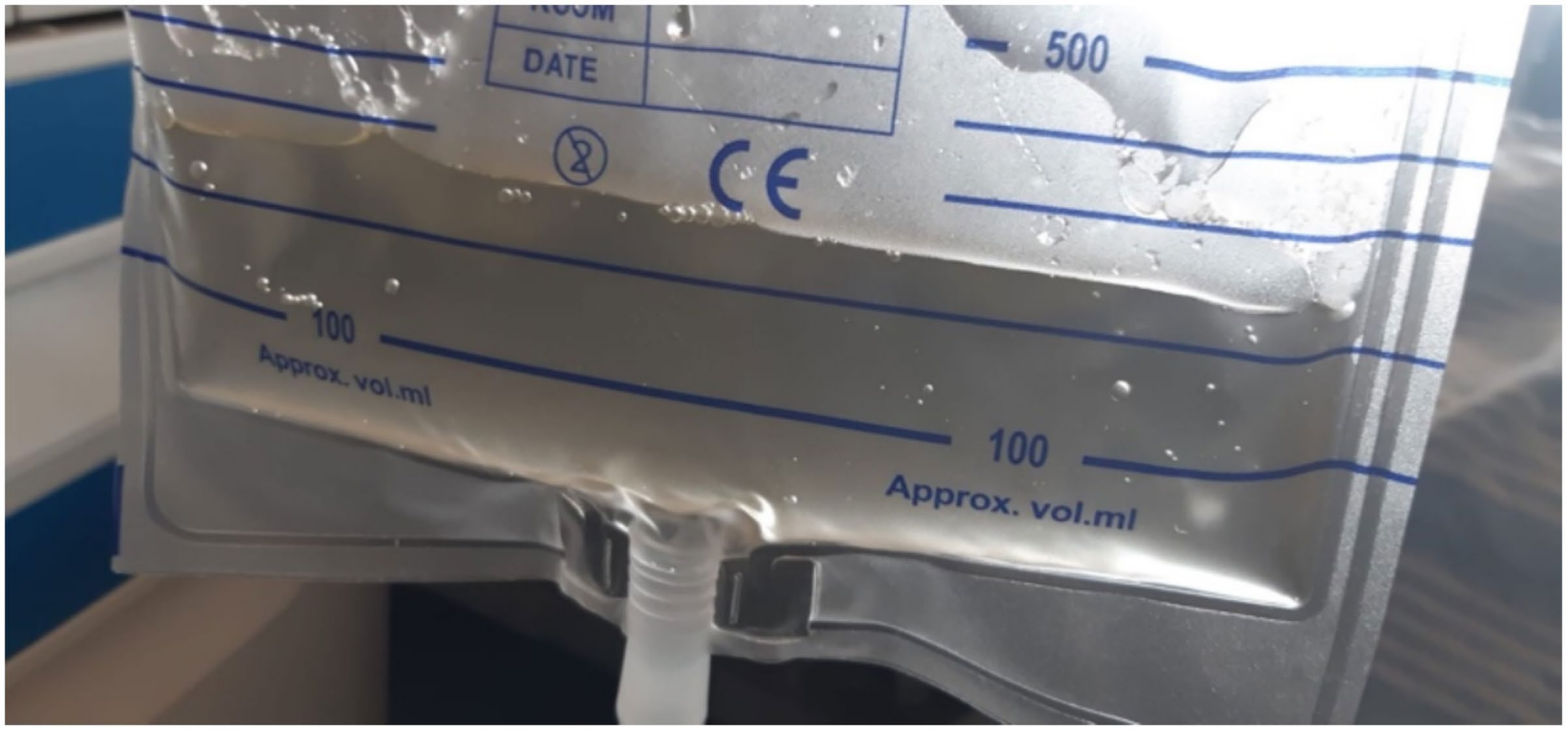
Pleural fluid appearance.

A non-contrast chest CT scan revealed a massive right-sided pleural effusion with almost complete atelectasis of the underlying lung. The patient underwent ultrasound-guided placement of a chest drain with pleural fluid notable for its appearance, closely resembling cerebrospinal fluid (Figure 1).

Biochemical analysis of the pleural fluid showed a markedly elevated glucose level (223 mg/dL) in the absence of hyperglycemia (serum glucose 86 mg/dL), very low protein content (0.3 g/dL), and low lactate dehydrogenase (35 U/L), consistent with a transudative effusion. Cytological examination ruled out malignant cells, and all microbiological cultures were negative. These findings raised clinical suspicion for a pleuroperitoneal leak of dialysis fluid.

**Figure 2:**
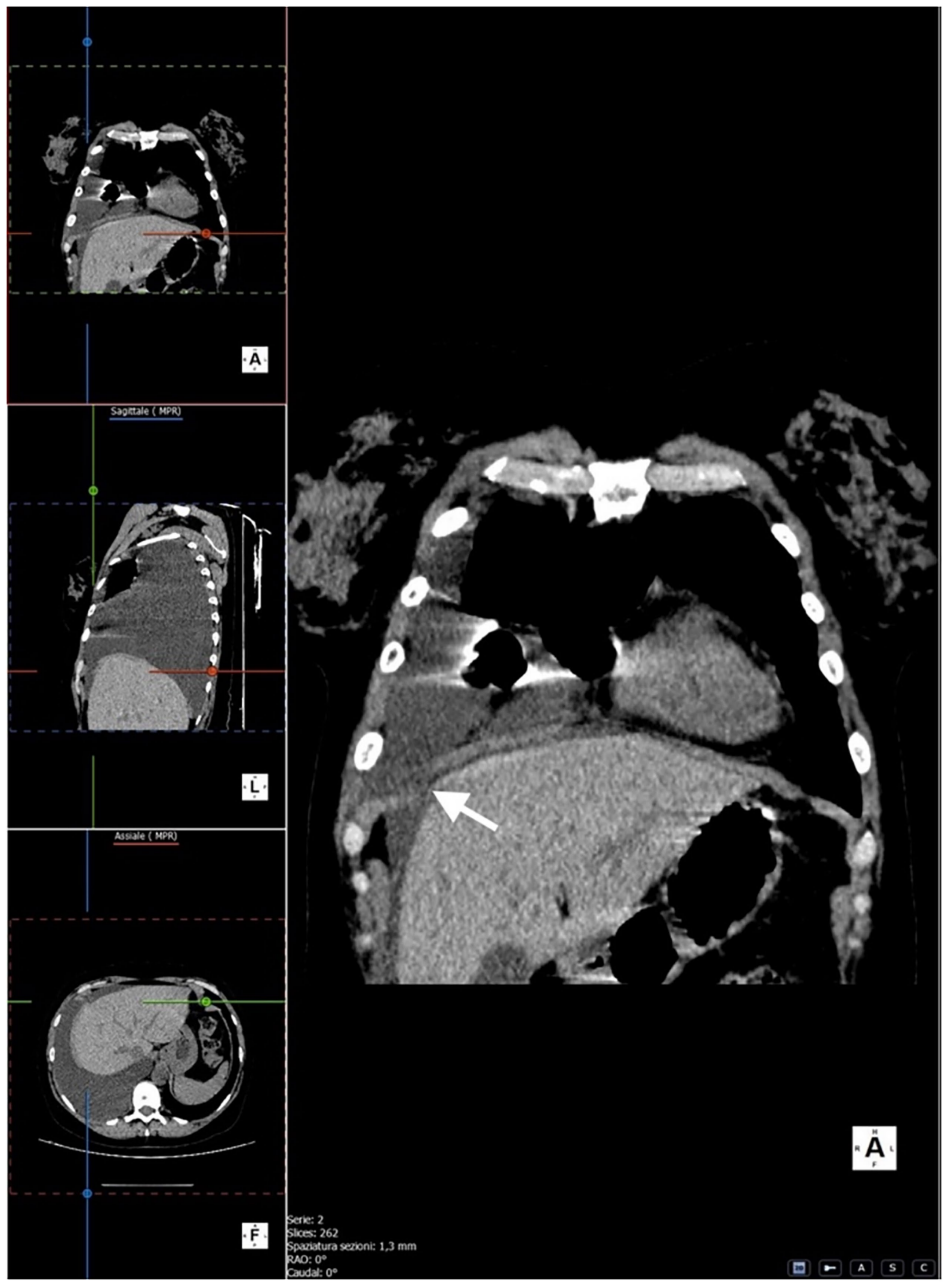
Coronal chest CT showing a suspected diaphragmatic discontinuity (white arrow).

**Figure 3:**
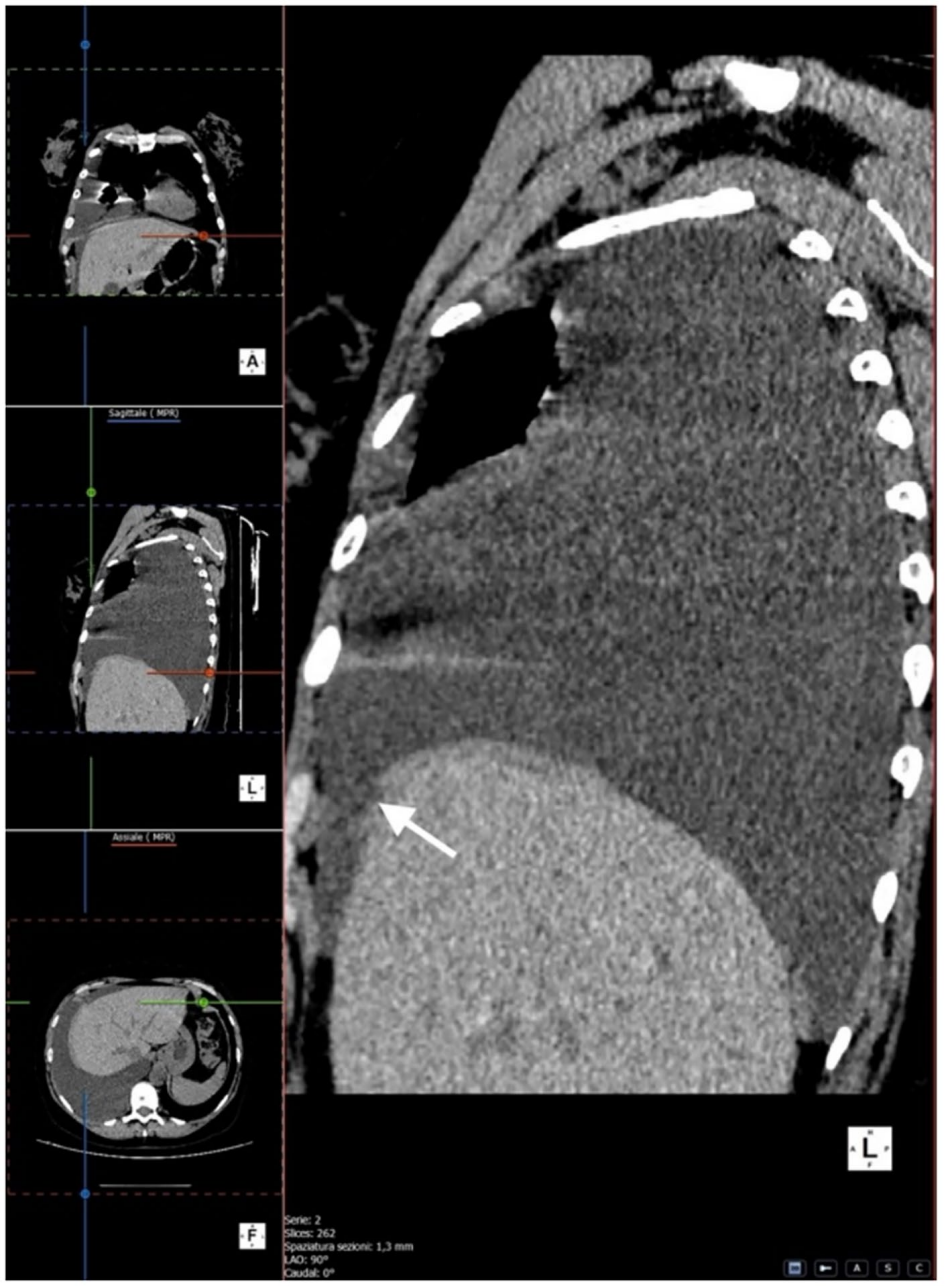
Sagittal CT reconstruction demonstrating a focal area suggestive of diaphragmatic discontinuity of the right hemidiaphragm (white arrow).

**Figure 4:**
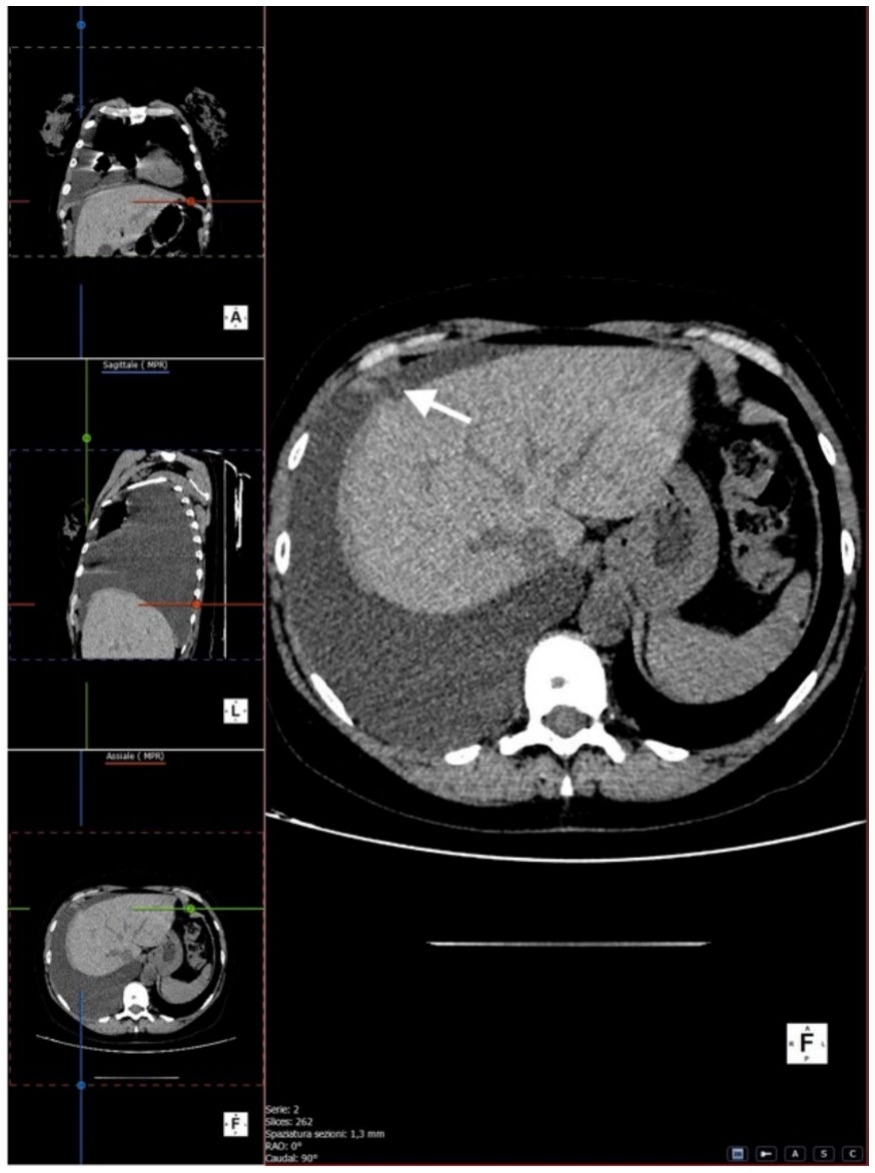
Axial CT images showing the same region of diaphragmatic irregularity (white arrow).

We performed multiplanar reconstruction of the chest CT, which revealed a focal area of suspected diaphragmatic discontinuity. However, the defect was not clearly visualized, and no direct evidence of dialysate passage across the diaphragm could be demonstrated. Therefore, imaging findings were considered suggestive but not diagnostic of pleuroperitoneal communication (Figures 2–4).

Peritoneal dialysis was immediately discontinued due to the strong clinical suspicion of PPC. The patient was transitioned to intermittent hemodialysis via central venous access as a renal replacement strategy. No additional pleural interventions were required after initial chest drainage. Five days after cessation of PD, bedside thoracic ultrasound showed complete resolution of the pleural effusion. The rapid resolution of the pleural effusion after discontinuation of PD was compatible with the suspected diagnosis and offered indirect clinical corroboration of pleuroperitoneal communication. Over the subsequent months and to date, the patient continued on HD without further complications.

The patient reported significant anxiety related to months of unexplained chronic pain. She described rapid symptomatic relief after discontinuation of peritoneal dialysis and expressed satisfaction with the diagnostic process and outcome.

Written informed consent for publication was obtained from the patient.

## Discussion

Pleural effusion represents a reported complication of peritoneal dialysis, and its occurrence has been associated with adverse clinical outcomes in dialysis populations, including increased morbidity and mortality (8). Pleural effusion typically presents with non-specific symptoms such as shortness of breath, cough, lower limbs edema, weight gain and reduced ultrafiltration volumes. Also 25% of patients remain completely asymptomatic (9). We described an atypical presentation with chronic and localized chest pain as the main symptom, in association with dyspnea. Wang et al. (4) also reported an unusual case presenting with an isolated syncopal episode and fatigue without the other commonly observed symptoms. In patients with end-stage renal disease, pleuroperitoneal communication must be differentiated from other common causes of pleural effusion, including fluid overload, congestive heart failure, and pneumonia. The pathophysiological mechanism involves a PPC caused by a defect in the diaphragm that allows the translocation of dialysate from a positive pressure district (abdomen) into a negative pressure district (pleural cavity). Pleural effusion due to diaphragmatic defect is usually an early complication of PD with 76% of reported cases occurring within 6 months and 89% within 12 months (4, 5). In a single-centre retrospective study, the mean duration of PD before symptoms onset was 177.0 ± 101.1 days (3). Our case occurred after approximately 12 months of PD, however a case has also been reported in which pleuroperitoneal leak presented after 15 months (6).

The variability in both clinical presentation and timing of onset, along with the lack of clearly defined diagnostic gold standards, makes this condition challenging to diagnose. Biochemical analysis of pleural fluid is a key diagnostic marker to differentiate a dialysate leak from other causes of transudative effusion. Chow et al. compared the biochemical analysis of pleural fluid in seven cases of pleural effusion secondary to pleuroperitoneal leak and found that a fluid-to-serum glucose gradient exceeding 50 mg/dL is highly sensitive of PPC (7). Alahmad et al. reported a specificity of 100% for this cut off value (10). Other reports have suggested that a pleural fluid–to–serum glucose ratio >1.0 may also be highly specific (11–13); however, variability in reported cases highlights the absence of a uniform biochemical pattern. Accordingly, no universally accepted diagnostic threshold has been established. For instance, Bohra et al. described a case in which the glucose gradient was <50 mg/dL with a fluid-to-serum glucose ratio >1.0 (14), but in a recent retrospective series, Cao et al. found that only 6 of 9 patients had a pleural fluid–to–serum glucose ratio >1, while 3 patients had a ratio <1 (3). Kennedy et al. (15) also reported a case in which biochemical analysis of pleural fluid did not reveal elevated glucose levels. In our case the pleural fluid was transudative according to Light’s criteria, with a pleural-to-serum glucose ratio of 2.59 and a glucose gradient of 137 mg/dL. The markedly elevated glucose level was highly suggestive of dialysate contamination, especially in the absence of hyperglycemia.

Radiological assessment can aid diagnosis but has low sensitivity. Chest X-ray can detect pleural effusion, but not the diaphragmatic defect. Radionuclide peritoneal scintigraphy typically performed using Tc-99 m–labeled agents, represents one of the most commonly employed diagnostic technique. It allows functional demonstration of dialysate migration from the peritoneal cavity into the pleural space and has reported sensitivities ranging from 40 to 50% (16–18). In one study this method could detect the presence of PPC in 70% of cases with majority within 12 minutes following contrast injection (5). However, while scintigraphy provides functional confirmation of pleuroperitoneal communication, it does not reliably localize the site of the diaphragmatic defect. For this reason and to improve diagnostic sensitivity, combined imaging approaches have been proposed. Ria et al. reported the use of peritoneal scintigraphy in association with CT peritoneography to achieve defect localization, which was subsequently confirmed during surgery (19). Newallo et al. reported the use of low-dose SPECT/CT integrated into peritoneal scintigraphy, performed using a low-volume (100 mL) normal saline and Tc-99 m sulfur colloid. In their case, SPECT/CT successfully detected the leakage whereas conventional scintigraphy did not, thereby improving sensibility (20).

Another option is CT peritoneography (CTP), performed after intraperitoneal instillation of iodinated contrast diluted in dialysate, which can provide combined functional and anatomical information and may aid diagnosis especially when the defect is large, with a reported sensitivity of 40–50% (21). Consistently, in a retrospective study, Cao et al. reported a sensitivity of 50% when CT was acquired 4 h after intraperitoneal contrast infusion (3). CTP has shown good sensitivity also in detecting atypical fistulous pathways, such as retroperitoneal routes (22); however, further systematic studies are needed to define its accuracy in localizing the diaphragmatic defect. Multiple technical factors may influence diagnostic sensitivity, including the intraperitoneal volume and pressure, patient position, and breath-holding during image acquisition; suboptimal conditions may reduce the transdiaphragmatic pressure gradient and result in failure to demonstrate the pleuroperitoneal leak (23, 24). In terms of procedural safety, CT peritoneography does not significantly affect residual renal function, with no meaningful changes in estimated glomerular filtration rate (eGFR) observed before and after the procedure in a recent retrospective study (3).

According to Prischl et al. magnetic resonance imaging (MRI) using only dialysate as the “contrast medium” can be another useful diagnostic tool; nevertheless, evidence remains limited and largely derives from case reports (25).

In our case, non-contrast chest CT with multiplanar reconstruction was performed to further investigate the suspected diaphragmatic defect. Multiplanar reconstructions (MPr) are routinely integrated into modern CT reporting workstations and can be performed without additional equipment, making them a readily accessible tool in routine clinical settings (26). However, CT provides only anatomical information and does not directly demonstrate active dialysate leakage. Accordingly, in our case, the imaging findings were only suggestive and not diagnostic of a diaphragmatic defect, as the discontinuity was not clearly visualized and no direct evidence of transdiaphragmatic passage could be demonstrated. In the absence of surgical exploration, the focal discontinuity observed on our multiplanar CT reconstruction cannot be conclusively established as the true site of PPC and the accuracy of defect localization cannot therefore be reliably determined. Similarly, Shah et al. also reported a case of pleuroperitoneal communication in which the diaphragmatic defect was visible on conventional chest CT; however, management was conservative, without surgical confirmation of the defect (27).

In addition to conventional imaging techniques, contrast-enhanced ultrasonography has recently been explored as a potential diagnostic adjunct. Inanaga et al. described the use of thoracic ultrasound following intraperitoneal instillation of dialysate mixed with 4.0 μL of perflubutane, in a case where CT peritoneography had failed to identify the leakage site (24). Although the ultrasound findings confirmed PPC, precise defect localization was not possible. Nevertheless, this technique is minimally invasive, low-cost, and widely available, suggesting that further studies are warranted to evaluate its potential role as an additional diagnostic tool in the workup of PPC.

Beyond imaging modalities, methylene blue instillation through the peritoneal dialysis catheter has been described as a simple diagnostic adjunct, allowing functional confirmation of pleuroperitoneal communication through observation of dye in pleural drainage (28, 29). In a recent report, Romero et al. employed this technique to confirm pleuroperitoneal communication; notably, the diaphragmatic fistula was also identifiable on CT imaging in the same case. However, management was conservative, and no surgical exploration was performed; therefore, confirmation of the diagnosis and precise localization of the fistula relied on response to treatment rather than intraoperative validation (28).

Evidence suggests that imaging accuracy is largely determined by defect size and position: larger defects are more likely to be identified, whereas smaller central tendon discontinuities—frequently described in operative reports (30)—may remain radiologically occult (24). However, the diagnosis of pleuroperitoneal communication should not rely on imaging findings alone but rather on the integration of clinical presentation, pleural fluid biochemical analysis and radiological assessment. In our case, the unilateral right-sided effusion in association with the elevated glucose concentration in pleural fluid provided strong evidence supporting the diagnosis. In this context, CT findings were interpreted as complementary anatomical information. The subsequent rapid resolution of the pleural effusion following discontinuation of peritoneal dialysis further supported the diagnostic coherence of this integrated approach.

Although CTP and radionuclide peritoneal scintigraphy remain the preferred diagnostic modalities due to their ability to provide functional confirmation of PPC, their availability may be limited in routine clinical practice; in contrast, CT with multiplanar reconstruction is widely accessible, easy and rapid to perform and can provide relevant anatomical clues. Moreover, in selected patients—particularly those who are not candidates for surgical repair or decline invasive procedures—functional confirmation may not be strictly required, and a presumptive diagnosis can be reasonably supported by the integration of CT findings with pleural fluid biochemistry, clinical context, and resolution after temporary discontinuation of peritoneal dialysis - as observed in our case. The present report does not aim to demonstrate a diagnostic superiority of CT over established functional imaging modalities, but rather to highlight its potential role as a readily available adjunct in selected clinical scenarios.

Management of pleuroperitoneal communication typically begins with prompt interruption of peritoneal dialysis and transition to hemodialysis for 2 to 6 weeks (7, 31). In our patient, this approach was chosen because of the patient’s preference to avoid surgical intervention and led to the complete and rapid resolution of the pleural effusion and the associated symptoms without the need for additional interventions. Temporary cessation of peritoneal dialysis may lead to spontaneous healing of the PPC in selected cases, especially in the presence of small defects, and may permit resumption of peritoneal dialysis. Nevertheless, the recurrence of this complication remains unpredictable, and continuation of PD, without surgical correction, has been rarely reported (7). The patient’s decision to resume or not peritoneal dialysis is a key factor in determining how to manage the PPC. In contemporary series, surgical management most commonly consists of video-assisted thoracoscopic surgery (VATS) with direct identification and repair of the diaphragmatic defect, using suturing, stapling, or patch reinforcement. This approach has been associated with high success rates, frequently exceeding 80–90%, and often allows safe resumption of peritoneal dialysis (14, 22, 29, 32–36). To improve intraoperative defect localization, several strategies have been described, including pneumoperitoneum-based approaches and intraperitoneal dye instillation. Indigo carmine and indocyanine green (ICG), sometimes combined with near-infrared imaging, have been employed during thoracoscopic repair to enhance detection of diaphragmatic defects and guide surgical closure (28, 30, 32, 37).

However, even VATS exploration may occasionally fail to localize the defect, reflecting the anatomical heterogeneity of PPC and the frequent presence of small or multiple fenestrations, which are typically described as minute, bleb-like lesions located within the central tendon of the diaphragm (23, 24, 30). Successful identification of the diaphragmatic defect appears to strongly correlate with surgical outcomes. Saito et al. (38) reported a success rate of 89% when the diaphragmatic defect was clearly identified and repaired, compared with only 38% when localization was unsuccessful. Accordingly, Inanaga et al. described a case in which CT peritoneography and intraoperative exploration with indigo carmine did not reveal the leakage site. The diaphragm was coated with polyglycolic acid sheets and fibrin glue; however, recurrence occurred 6 months later, further emphasizing the prognostic importance of precise defect localization (24).

In addition, pleurodesis represents another established therapeutic option, with a reported efficacy of approximately 50% (39). Chemical pleurodesis may be performed either through thoracic drainage or during VATS, using agents such as talc, hypertonic glucose, or autologous blood. This strategy aims to obliterate the pleural space and prevent recurrent dialysate leakage, particularly in cases where the defect cannot be clearly identified intraoperatively. Cao et al. described intrapleural elemene infusion, administered via the thoracic drainage tube after complete evacuation of the pleural effusion in a cohort of 12 patients, with recurrence observed in only three cases during follow-up (3). Wang et al. reported a case managed with VATS and direct repair of the diaphragmatic defect combined with pleurodesis and subsequent *Pseudomonas aeruginosa* instillation into the pleural cavity in order to promote pleural adhesion. No perioperative complications were described, peritoneal dialysis was successfully resumed, and no recurrence was observed during follow-up (29). However, these approaches have been reported only in isolated reports and require further systematic evaluation before being considered standard therapeutic options.

## Conclusion

Pleuroperitoneal communication is an uncommon but important cause of pleural effusion in PD patients. Clinical presentation may be variable and occasionally atypical, as in our case, where chronic localized chest pain accompanied dyspnea. Diagnosis requires an integration of clinical, biochemical, and radiological data.

Although chest CT with multiplanar reconstruction cannot provide functional confirmation of active leakage, in our experience it may offer useful anatomical findings suggestive of diaphragmatic discontinuity and contribute to the overall diagnostic assessment, particularly when interpreted within a strongly supportive biochemical and clinical context. Definitive localization of the defect, however, remains dependent on surgical confirmation.

Early recognition of PPC and prompt interruption of peritoneal dialysis are essential to achieve rapid resolution of pleural effusion. However, current evidence suggests that VATS with direct repair of the diaphragmatic defect has the highest efficacy in preventing recurrence and enabling safe resumption of peritoneal dialysis.

## Data Availability

The original contributions presented in the study are included in the article/Supplementary material, further inquiries can be directed to the corresponding author.

## References

[1] Mahmoud HAM Mahmoud AA Mobark IS Hassan AT. Pleural complications in patients with chronic and end-stage renal disease. Eur Respir J. (2014) 44:P597. doi: 10.1183/13993003/erj.44.Suppl_58.P597

[2] Nomoto Y Suga T Nakajima K Sakai H Osawa G Ota K . Acute hydrothorax in continuous ambulatory peritoneal dialysis—a collaborative study of 161 centers. Am J Nephrol. (1989) 9:363–7. doi: 10.1159/000167997 2679094

[3] Cao P Su K Li F Wang X Yuan W Lu S . Efficacy and safety of pleural infusion of elemene in the treatment of peritoneal dialysis complicated with pleuroperitoneal communication. Wien Klin Wochenschr. (2025). doi: 10.1007/s00508-025-02683-8, 41428218

[4] Wang A Hu SL Shah AD. Hydrothorax with atypical features as a complication of peritoneal dialysis: a case report and literature review. Brown J Hosp Med. (2023) 2:57690. doi: 10.56305/001c.57690 40046538 PMC11878848

[5] Harry L Nyakale N Tinarwo P. Scintigraphic peritoneography in the diagnosis of pleuroperitoneal leak complicating peritoneal dialysis. Medicine. (2020) 99:e21029. doi: 10.1097/MD.0000000000021029 32769864 PMC7593027

[6] Kwan VSY Leibowitz S. Delayed pleuroperitoneal leak in an otherwise uncomplicated course of peritoneal dialysis. Clin Case Reports. (2023) 11:e7469. doi: 10.1002/ccr3.7469 PMC10264940 37323290

[7] Chow KM Szeto CC Wong TYH Li PKT. Hydrothorax complicating peritoneal dialysis: diagnostic value of glucose concentration in pleural fluid aspirate. Perit Dial Int. (2002) 22:525–7. doi: 10.1177/089686080202200416, 12322829

[8] Zhang F Xiang T Feng X Zhang G Liu Y Li L. Pleural effusion portends a poor prognosis in patients on continuous ambulatory peritoneal dialysis. PLoS One. (2024) 19:e0297343. doi: 10.1371/journal.pone.0297343, 38241413 PMC10798541

[9] Bae EH Kim CS Choi JS Kim SW. Pleural effusion in a peritoneal dialysis patient. Chonnam Med J. (2011) 47:43–4. doi: 10.4068/cmj.2011.47.1.43, 22111056 PMC3214860

[10] Mohamad Alahmad MA Kasmani R. Sweet hydrothorax: a common presentation of a rare condition. Avicenna J Med. (2019) 9:111–4. doi: 10.4103/ajm.ajm_131_18, 31404134 PMC6647920

[11] Tang S Chui WH Tang AWC Li FK Chau WS Ho YW . Video-assisted thoracoscopic talc pleurodesis is effective for maintenance of peritoneal dialysis in acute hydrothorax complicating peritoneal dialysis. Nephrol Dial Transplant. (2003) 18:804–8. doi: 10.1093/ndt/gfg042, 12637652

[12] Mangana P Arvanitis D Vlassopoulos D. Acute hydrothorax in peritoneal dialysis patients: diagnosis and treatment options. Nephrol Dial Transplant. (2003) 18:2451. doi: 10.1093/ndt/gfg408 14551387

[13] Momenin N Colletti PM Kaptein EM. Low pleural f luid-to-serum glucose gradient indicates pleuroperitoneal communication in peritoneal dialysis patients: presentation of two cases and a review of the literature. Nephrol Dial Transplant. (2012) 27:1212–9. doi: 10.1093/ndt/gfr393 21771760

[14] Bohra N Sullivan A Chaudhary H Demko T. The use of pleural fluid to serum glucose ratio in establishing the diagnosis of a not so sweet PD-related hydrothorax: case report and literature review. Case Rep Nephrol. (2020) 2020:1–4. doi: 10.1155/2020/8811288, 32963857 PMC7492928

[15] Kennedy C McCarthy C Alken S McWilliams J Morgan RK Denton M . Pleuroperitoneal leak complicating peritoneal dialysis: a case series. Int J Nephrol. (2011) 2011, 2011:1–4. doi: 10.4061/2011/526753, 21876802 PMC3161202

[16] Cho Y D’Intini V Ranganathan D. Acute hydrothorax complicating peritoneal dialysis: a case report. J Med Case Rep. (2010) 4:355. doi: 10.1186/1752-1947-4-355, 21059200 PMC2987964

[17] Álvarez Mena N Gamazo Laherrán C Pérez López B Alonso Rodríguez M Ruiz Gómez MA Ruano Pérez R. Confirmation of the pleuroperitoneal leak origin with a SPECT-CT in a patient on peritoneal dialysis. Rev Esp Med Nucl Imagen Mol (Engl Ed). (2020) 39:381–2. doi: 10.1016/j.remnie.2020.06.010 32546412

[18] Chavannes M Sharma AP Singh RN Reid RH Filler G. Diagnosis by peritoneal scintigraphy of peritoneal dialysis-associated hydrothorax in an infant. Perit Dial Int. (2014) 34:140–3. doi: 10.3747/pdi.2012.00077 24525610 PMC3923712

[19] Ria P Luongo L Martella V Zito A Matino S Barbarini S . Advanced strategies for managing Pleuroperitoneal communication in peritoneal Dialysis patients: report of two cases. Am J Case Rep. (2025) 26:e947860. doi: 10.12659/AJCR.947860, 40751305 PMC12327152

[20] Newallo DS Chataigne M Muzahir S. The role of SPECT/CT in peritoneal scintigraphy in the era of low-dose imaging: a case report. World J Nucl Med. (2022) 21:065–8. doi: 10.1055/s-0042-1748030, 35502280 PMC9056127

[21] Ambarsari CG Bermanshah EK Putra MA Rahman FHF Pardede SO. Effective Management of Peritoneal Dialysis-Associated Hydrothorax in a child: a case report. Case Rep Nephrol Dial. (2020) 10:18–25. doi: 10.1159/000506119, 32232056 PMC7098331

[22] Nakamura A Morimoto K Yoshida T. Pleuroperitoneal communication through the retroperitoneum. Clin Exp Nephrol. (2025) 29:505–6. doi: 10.1007/s10157-024-02613-4, 39719544

[23] Nakayama T Hashimoto K Kiriyama T Hirano K. Optimal imaging conditions for the diagnosis of pleuroperitoneal communication. BMJ Case Rep. (2019) 12:e228940. doi: 10.1136/bcr-2018-228940, 30936360 PMC6453368

[24] Inanaga R Oda M Asahina K Muraki N Jimbo M Shiga K . The new method to make diagnosis and identify the location of leakage of pleuroperitoneal communication in peritoneal dialysis patients. CEN Case Rep. (2022) 11:471–6. doi: 10.1007/s13730-022-00701-6, 35428968 PMC9626705

[25] Prischl FC Muhr T Seiringer EM Funk S Kronabethleitner G Wallner M . Magnetic resonance imaging of the peritoneal cavity among peritoneal Dialysis patients, using the dialysate as “contrast medium”. J Am Soc Nephrol. (2002) 13:197–203. doi: 10.1681/ASN.V131197 11752038

[26] Faggioni L Neri E Cerri F Turini F Bartolozzi C. Integrating image processing in PACS. Eur J Radiol. (2011) 78:210–24. doi: 10.1016/j.ejrad.2009.06.022 19619971

[27] Shah M Mcnamara B Abdel-Rahman EM. Early-onset dyspnea in a patient initiating peritoneal dialysis. Kidney360. (2024) 5:929–30. doi: 10.34067/KID.0000000000000467, 38935494 PMC11219100

[28] García Romero JM Guerrero Morales PH Alegria Arias AL de Noriega GD Bulle PM. Methylene blue instillation: a cost-effective diagnostic approach for pleuroperitoneal fistula in resource-limited settings. Cureus. (2024) 16:e69034. doi: 10.7759/cureus.69034, 39391407 PMC11464615

[29] Wang L Liu J Wang Y Zhu L Hu J. Minimally invasive surgery for pleuroperitoneal communication complicating peritoneal dialysis. Int Urol Nephrol. (2023) 55:3189–95. doi: 10.1007/s11255-023-03585-2 37072602

[30] Hashimoto T Osaki T Oka S Fujikawa T. Thoracoscopic and laparoscopic approach for pleuroperitoneal communication under peritoneal dialysis: a report of four cases. Surg Case Rep. (2023) 9:55. doi: 10.1186/s40792-023-01635-6, 37029287 PMC10082140

[31] Puri V Orellana FA Singer GG Wald MS. Diaphragmatic defect complicating peritoneal dialysis. Ann Thorac Surg. (2011) 92:1527. doi: 10.1016/j.athoracsur.2011.03.120 21958814

[32] Suyama Y Kinoshita T Otani A Tsukamoto Y Shibazaki T Nakada T . A new intraoperative method for detecting fistulas in patients with peritoneal Dialysis-associated Pleuroperitoneal communication. Ann Thorac Surg. (2025) 3:943–5. doi: 10.1016/j.atssr.2025.04.011, 41425417 PMC12712213

[33] Dang MH Mathew M Raj R. Pleuroperitoneal leak as an uncommon cause of pleural effusion in peritoneal Dialysis: a case report and literature review. Case Rep Nephrol. (2020) 2020:1–4. doi: 10.1155/2020/8832080, 32934854 PMC7479454

[34] Jonny J Violetta L. Bilateral pleural effusion in continuous ambulatory peritoneal dialysis managed by VATS pleurodesis. Eur J Case Rep Intern Med. (2024) 11:004343. doi: 10.12890/2024_004343, 38584902 PMC10997387

[35] Lafrid M Labioui N El Hammoumi MM Hallak M Laasli H Bahadi A . Pleuroperitoneal leak: a rare complication of peritoneal dialysis. Biomed Hub. (2025) 10:93–7. doi: 10.1159/000545281, 40352969 PMC12064151

[36] Matsuoka N Yamaguchi M Asai A Kamiya K Kinashi H Katsuno T . The effectiveness and safety of computed tomographic peritoneography and video-assisted thoracic surgery for hydrothorax in peritoneal dialysis patients: a retrospective cohort study in Japan. PLoS One. (2020) 15:e0238602. doi: 10.1371/journal.pone.0238602, 32881941 PMC7470296

[37] Kou Y Yamazaki N Sakaguchi Y Tanaka H Sonobe M. A new technique to detect communication sites for pleuroperitoneal communication. Gen Thorac Cardiovasc Surg. (2022) 70:591–2. doi: 10.1007/s11748-022-01777-8 35113316

[38] Saito M Nakagawa T Tokunaga Y Kondo T. Thoracoscopic surgical treatment for pleuroperitoneal communication. Interact Cardiovasc Thorac Surg. (2012). 15, 788–9. doi: 10.1093/icvts/ivs193 22753435 PMC3445346

[39] Mak SK Nyunt K Wong PN Lo KY Tong GM Tai YP . Long-term follow-up of thoracoscopic pleurodesis for hydrothorax complicating peritoneal dialysis. Report. (2002) 74:218–21. doi: 10.1016/S0003-4975(02)03648-2 12118762

[40] Hojs R Piko N Hren M Bevc S Ekart R. A case of ‘sweet’ hydrothorax in a patient on peritoneal Dialysis. Eur J Case Rep Intern Med. (2019) 6:1. doi: 10.12890/2019_001060, 30931279 PMC6438108

[41] Bhattarai K Acharya A Sarkar S. Peritoneal leak causing right sided hydrothorax in a case of chronic kidney disease undergoing continuous ambulatory peritoneal Dialysis: a case report from Nepal. Clin Case Reports. (2024) 12:e9577. doi: 10.1002/ccr3.9577, 39555206 PMC11564126

